# The Role of Long Non-Coding RNA and microRNA Networks in Hepatocellular Carcinoma and Its Tumor Microenvironment

**DOI:** 10.3390/ijms221910630

**Published:** 2021-09-30

**Authors:** Tingting Shi, Asahiro Morishita, Hideki Kobara, Tsutomu Masaki

**Affiliations:** Department of Gastroenterology and Neurology, Faculty of Medicine, Kagawa University, 1750-1 Ikenobe, Miki, 761-0793, Japan; asahiro@med.kagawa-u.ac.jp (A.M.); kobara@med.kagawa-u.ac.jp (H.K.)

**Keywords:** hepatocellular carcinoma, microRNA, long non-coding RNA, tumor microenvironment, biological function

## Abstract

Hepatocellular carcinoma (HCC) is a common liver malignancy with high morbidity and poor prognosis. Long non-coding RNAs (lncRNAs) are involved in crucial biological processes of tumorigenesis and progression, and play four major regulatory roles, namely signal, decoy, guide, and scaffold, to regulate gene expression. Through these processes, lncRNAs can target microRNAs (miRNAs) to form lncRNA and miRNA networks, which regulate cancer cell proliferation, metastasis, drug resistance, and the tumor microenvironment. Here, we summarize the multifaceted functions of lncRNA and miRNA networks in the pathogenesis of HCC, the potential use of diagnostic or prognostic biomarkers, and novel therapeutic targets in HCC. This review also highlights the regulatory effects of lncRNA and miRNA networks in the tumor microenvironment of HCC.

## 1. Introduction

Hepatocellular carcinoma (HCC) is a common liver malignancy with high morbidity and poor prognosis. Recent global cancer statistics indicate that primary liver cancer is the sixth most commonly diagnosed cancer and the third leading cause of cancer death with approximately 906,000 new cases and 830,000 deaths (8.3%) occurring in 2020 [1]. HCC constitutes 75–85% of primary liver cancers. Risk factors, including hepatitis virus (hepatitis B virus, HBV or hepatitis C virus, HCV) infection, alcohol abuse, metabolic liver disease, type 2 diabetes, and exposure to dietary toxins (aflatoxins and aristolochic acid), vary from region to region [1,2]. These risk factors provoke an inflammatory response and liver injury, which promotes liver fibrosis, epigenetic changes during hepatocyte renewal, and microenvironment changes [3,4]. These changes enhance liver damage, and chronic positive feedback accelerates tumorigenesis [3,4]. The strategy of HCC treatment depends on the stage of HCC: For early stage, ablation, resection, and liver transplantation could be curative therapy; transarterial chemoembolization (TACE), transarterial embolization (TAE), transarterial radioembolization (TARE), and systemic treatment (sorafenib, lenvatinib [5] as first-line therapy) could be used for intermediate-stage HCC; for advanced-stage, systemic treatment with multi-kinase inhibitors or immune checkpoint inhibitors could prolong the overall survival (OS) rates [2]. However, the current 5-year cause-specific survival rate remains low [6]. Thus, early diagnosis and intervention play a key role in improving HCC survival.

MicroRNAs (miRNAs) and long non-coding RNAs (lncRNAs) are non-coding RNAs. miRNAs are approximately 17–25 nucleotides long and their major biological function is regulating target gene expression by binding to the 3′-UTR of target mRNAs, which is associated with cell proliferation, cell death, and signaling pathway regulation [7]. Furthermore, miRNAs with similar sequences are organized in clusters, leading to combinatorial diversity and synergy in biological functions [8]. In humans, approximately 30% of genes are regulated by miRNAs, and miRNA regulation is closely associated with various diseases, particularly cancers [9].

lncRNAs, which are over 200 nucleotides in length, do not translate into proteins; however, their secondary structures or three-dimensional structures enable them to have protein-like functions [10,11]. lncRNAs are involved in diverse biological processes via binding to DNA, RNA, and proteins [12] and play four main regulatory roles, including signal, decoy, guide, and scaffold, to regulate gene expression [13]. The functions of lncRNAs include the modification of chromatin through lncRNA–protein or lncRNA–DNA interactions; the activation or repression of transcription by interaction with transcriptional coactivators or acting as a decoy to keep transcriptional activators away; translation control of proteins; the stabilization of mRNA; the degradation of mRNA; playing the role of competing endogenous RNA (ceRNA) by interacting with miRNAs; miRNA production; and the regulation of the phosphorylation, methylation, and ubiquitination of mRNA and protein modifications [14]. In addition, the intercellular transfer of non-coding RNAs through extracellular vesicles (EVs), such as miRNAs, can modulate cellular function even in the microenvironment [15].

Recently, an increasing number of studies have focused on the emerging roles of lncRNAs and miRNAs in HCC and the tumor microenvironment [16,17,18]. In this review, the lncRNA and miRNA networks in HCC and the tumor microenvironment have been discussed. We summarize the multifaceted functions of lncRNA and miRNA networks underlying the pathogenesis of HCC, the potential use of diagnostic or prognostic biomarkers, and novel therapeutic targets in HCC. 

## 2. Biological Functional Relationship between lncRNAs and miRNAs

**Figure 1:**
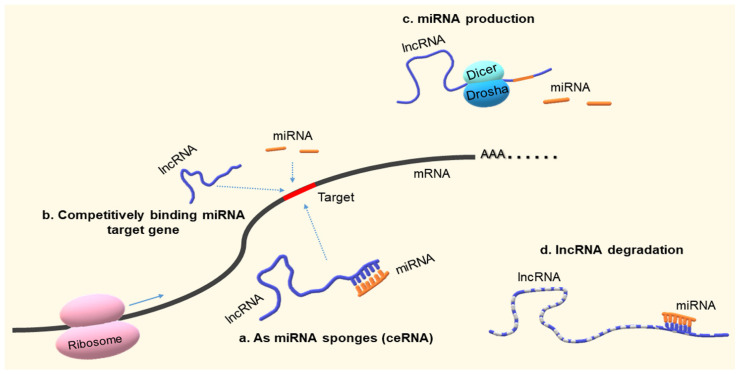
The biological functional relationship between long non-coding RNA and microRNA. lncRNA, long non-coding RNA; miRNA, microRNA; ceRNA, competing endogenous RNA.

The biological functional relationship between lncRNA and miRNA may be briefly summarized as miRNA sponges (ceRNA), competitive binding to miRNA target genes, miRNA production from lncRNAs, and lncRNA degradation. In detail, lncRNAs act as ceRNAs by binding target miRNAs to prevent the binding of miRNAs to their target genes, upregulating target gene expression, which functions as the lncRNA–miRNA–mRNA axis. To date, most studies have focused on this interaction axis. In addition, lncRNAs can also bind to sites on the target genes of miRNAs to regulate their corresponding miRNA activity [14,19]. lncRNAs, which contain miRNA sequences, can produce miRNAs by Dicer and/or Drosha cleavage and regulate different points of miRNA biogenesis, affecting microprocessor activity to finish the primary transcript independent of polyadenylation [20]. miRNAs can bind target lncRNAs to regulate the stability and half-life of lncRNAs [20,21]. These complex interactions between lncRNAs and miRNAs constitute the lncRNA and miRNA networks, mainly through sequence complementarity (Figure 1).

## 3. Role of lncRNA and miRNA Networks in HCC

The interaction and regulation of lncRNAs and miRNAs can have a direct effect on the corresponding target genes and regulate the translation of proteins, which play an important role in tumorigenesis and the progression of several types of cancers, including HCC, mainly through the lncRNA–miRNA–mRNA axis. During the development and progression of cancers, some miRNAs, which act as oncogenic miRNAs, are upregulated, while others, which act as tumor suppressors, are downregulated [22]. Thus, in the lncRNA and miRNA networks, dominating lncRNAs that bind to oncogenic miRNAs act as suppressors; on the contrary, those that bind to inhibitor miRNAs play the role of promoters (Table 1). 

### 3.1. Promoting Effect of lncRNA and miRNA Networks in HCC

Studies of the lncRNA–miRNA–mRNA axis are majorly represented in the lncRNA and miRNA networks. The lncRNA myocardial infarction-associated transcript (MIAT) is overexpressed in HCC tissues and cells, promotes HCC cell proliferation and invasion by directly sponging miR-214, and upregulates the expression of the enhancer of zeste homolog 2 (EZH2) and β-catenin [23]. EZH2 acts as a key regulator of cell-cycle progression, promotes DNA damage repair, and accelerates cell proliferation that together contribute to carcinogenesis and cancer progression [68]. Additionally, EZH2 is overexpressed in HCC and is associated with a poor prognosis [69]. β-catenin is a key component of Wnt signaling, and its expression can be upregulated in cancer by stabilizing mutations, which stimulate WNT/β-catenin signaling. Dysregulation of the WNT/β-catenin pathway is related to HCC, while up to 66% of HCC cases have aberrant WNT/β-catenin signaling [70]. MIAT also acts as a ceRNA of miR-22-3p to upregulate the expression of sirt1 in HCC cells. Additionally, decreased MIAT activates the p53/p21 and p16/pRb pathways, which suppresses HCC cell proliferation [24]. The lncRNA HOXD cluster antisense RNA 1 (HOXD-AS1) is upregulated in HCC tissues and is positively associated with poor prognosis and node metastasis in HCC patients. HOXD-AS1 competitively sponges miR-130a-3p, which prevents Sry-related HMG box-4 (SOX4) degradation and activates EZH2 and matrix metalloproteinase-2 (MMP-2) protein expression to promote HCC metastasis [26]. HOXD-AS1 also upregulates the levels of Rho GTPase activating protein 11A (ARHGAP11A) by targeting miR-19a, which induces metastasis and reduces apoptotic effects [27]. Moreover, the silencing of HOXD-AS1 inhibits HCC cell proliferation, cell cycle progression, and invasion via the MEK/ERK pathway [71]. 

A clinical study including 129 HCC patients showed that the levels of serum lncRNA were highly upregulated in liver cancer (HULC); metastasis-associated lung adenocarcinoma transcript 1 (MALAT1), Linc00152, pituitary tumor-transforming 3 pseudogene (PTTG3P), SPRY4 intronic transcript 1 (SPRY4-IT1), ubiquitin-conjugating enzyme E2C pseudogene 3 (UBE2CP3), and urothelial carcinoma-associated 1 (UCA1) were significantly higher in HCC patients than in benign liver disease patients and healthy controls [72]. LncRNA dysregulation is also associated with carcinogenesis and could be used to predict HCC diagnosis and prognosis. The binding of lncRNA HULC to miR-6825-5p, miR-6845-5p, and miR-6886-3p upregulates ubiquitin-specific peptidase 22 (USP22) expression to stabilize Sirt1, which induces HCC cell autophagy. In addition, chemotherapeutic agents, such as 5-fluorouracil and pirarubicin (THP), induce HULC expression and protective autophagy in HCC cells [28]. Furthermore, HULC could enhance the stability of HBV covalently closed circular DNA (cccDNA) and activate HBV replication in HBV-positive HCC cells [73]. HULC also enhanced the Epithelial–mesenchymal transition (EMT) by acting as a ceRNA and binding to miR-200a-3p and increasing the expression of zinc finger E-box binding homeobox 1 (ZEB1), promoting tumorigenesis and metastasis [74]. MALAT1 sponges miR-125a-3p to upregulate forkhead box M1 (FOXM1) expression, which promotes HCC cell proliferation [54]. Moreover, MALAT1 facilitates HCC development by regulating glucose metabolism, enhancing glycolysis, and inhibiting gluconeogenesis [75]. The binding of lncRNA UCA1 to miR-216b upregulates the expression of fibroblast growth factor receptor 1 (FGFR1) to activate the ERK signaling pathway, which in turn promotes HCC progression [76].

### 3.2. Inhibitory Effect of lncRNA and miRNA Networks in HCC

The downregulation of lncRNA cancer susceptibility candidate 2 (CASC2) in HCC tissues and HCC cells and binding of CASC2 to miR-367 to upregulate F-box/WD repeat-containing protein 7 (FBXW7) expression inhibits EMT in HCC [56]. Moreover, upregulation of CASC2 could improve cisplatin sensitivity by sponging miR-222 directly [77]. lncRNA MAGI2 antisense RNA 3 (MAGI2-AS3) has been found to be downregulated in HCC tissues and is negatively associated with lymph node metastasis, TNM stage, and overall survival. Upregulated MAGI2-AS3 directly binds to miR-374b-5p and positively regulates the expression of the suppressor with morphogenetic effects on genitalia family member 1 (SMG1) in HCC, which inhibits HCC cell proliferation and migration [60]. The lncRNA DiGeorge syndrome critical region gene 5 (DGCR5) was downregulated in HCC tissues and serum, which correlated closely with poor cancer-specific survival, with an overall 5-year rate of 10.3% in the low expression group and 36.6% in the high expression group, respectively [78]. Additionally, DGCR5 was found to act as a sponge of miR-346 to modulate the expression of Krüppel-like factor 14 (KLF14) and inhibit the development of HCC [62]. 

The lncRNA MIR22HG is associated with the initiation and progression of many human cancers. MIR22HG was reported to decrease in HCC tissues and predict the poor prognosis of HCC patients, as it functions as a tumor suppressor and inhibits cell proliferation and invasion by sponging miRNA-10a-5p to upregulate nuclear receptor co-repressor 2 (NCOR2) expression [67]. miR10a-5p is reported to promote HCC cell growth, migration, and invasion [67]. NCOR2 can disrupt the binding of β-catenin to the transducin-beta-like protein 1 (TBL1)/transducing-beta-like 1 X-linked receptor 1 (TBLR1) complex to inactivate the Wnt/β-catenin pathway, thereby suppressing EMT [79]. Moreover, MIR22HG suppresses the proliferation and metastasis of cancer cells also through MIR22HG-derived miR-22-3p targeting high mobility group box 1 (HMGB1) and binding to human gene antigen R (*HuR*) [80]. HMGB, a damage-associated molecule, is a key tumor promoter in HCC. *HuR* can stabilize oncogenic mRNAs, such as *CTNNB1*, *CCNB1*, *HIF1A*, *BCL2*, and *COX2*, while MIR22HG preferentially binds to *HuR* and decreases the expression of these oncogenes, thereby destabilizing many oncogenes [80]. A recent study demonstrated that irradiation downregulates histone deacetylase (HDAC)-2 expression and upregulates the expression of MIR22HG, which promotes the production of miR-22-5p. Overexpression of MIR22HG and miR-22-5p increases the sensitivity of HCC to radiotherapy [81]. 

### 3.3. LncRNAs and microRNAs May Act as New Biomarkers in HCC Diagnosis and Prognosis

Patients with HCC risk factors, such as HBV/HCV infection and cirrhosis, who were enrolled in a surveillance program showed a reduced HCC-related mortality rate and an increased early-stage diagnosis rate compared with unenrolled patients, although the survival benefit in patients with cirrhosis remains controversial [82]. The best-studied and most-used biomarker yet remains alpha fetoprotein (AFP), although it has been demonstrated to have poor sensitivity for HCC when used alone. A retrospective case-control study revealed that the sensitivity was only approximately 60% and the specificity was 80%, even considering the most efficient cutoff (10–20 ng/mL) [83]. To increase detection sensitivity, it is important to minimize false-positive results of AFP. However, a meta-analysis study indicated that AFP with ultrasound detected early-stage HCC with 63% sensitivity (95% CI, 48–75%), while that without ultrasound detected early-stage HCC with 45% sensitivity (95% CI, 30–62%) [84]. Moreover, a randomized controlled surveillance trial including 18,816 HBV patients who underwent an AFP test and an ultrasonography examination every six months indicated that the mortality rate was significantly lower in the screened group (83.2/100,000) than in controls (131.5/100,000) [85]. From an economic point of view, AFP testing with ultrasonography examination is a good option for HCC surveillance but it currently remains insufficient as a biomarker. Serum biomarkers are an attractive choice for the surveillance, early diagnosis, and prognosis of HCC because they allow non-invasive, objective, and reproducible evaluation [83]. Therefore, there is increasing interest in serum biomarkers that may improve sensitivity for the early detection of HCC (Table 2 and Table 3).

Recent studies suggest that lncRNA may act as a new biomarker for HCC diagnosis and prognosis (Table 2). The levels of plasma lncRNA zinc finger antisense 1 (ZFAS1) were significantly higher in HCC patients than those in healthy controls (AUC= 0.801, 95% CI: 0.724–0.875), with a sensitivity and specificity of 55.7% and 90.0%, respectively. Moreover, a combination of AFP and ZFAS1 increased the diagnostic efficiency (AUC= 0.891 95% CI: 0.829–0.953) [86]. Serum extracellular vesicle-derived lncRNA LINC00853 was increased in HCC patients and showed obvious discriminatory ability in the diagnosis (AUC = 0.934, 95% CI: 0.887–0.966) with a sensitivity of 93.75% and specificity of 89.77%. At the same time, serum EV-derived LINC00853 showed 97% positivity in AFP-negative HCC patients [87]. The expression of lncRNA tetraspanin-12 (TSPAN12) was upregulated in HCC with microvascular invasion (MVI) (AUC = 0.855, 95% CI: 0.797–0.912) with a sensitivity and specificity of 76.3% and 80%, respectively. Furthermore, TSPAN12 is an independent prognostic predictor for overall survival (HR = 2.924 95% CI: 1.460–5.855) [88]. In HBV-related HCC patients, the levels of lncRNA semaphorin 6A-antisense RNA 1 (SEMA6A-AS1) were reduced, correlated negatively with AFP levels, and were significantly associated with poor overall survival [89]. The levels of serum Linc00152 and UCA1 were significantly increased in HCC patients, and the panel of serum linc00152, UCA1, and AFP showed significant predictive ability, with an AUC of 0.912 and sensitivity and specificity of 82.9% and 88.2%, respectively [72]. Serum lncRNA NONHSAT053785 levels, which were significantly upregulated in HCC patients (AUC = 0.801), were higher in HCC patients with intrahepatic metastasis (AUC = 0.678). In addition, NONHSAT053785 could act as an independent predictor of intrahepatic metastasis in the elderly, drinking or non-smoking patients, and patients with tumor size > 5 cm [96].

Furthermore, circulating miRNAs have also been reported as potential novel biomarkers for HCC diagnosis and prognosis (Table 3). Expression levels of serum miR-21 were significantly higher in HCC patients than in controls with an AUC of 0.849, a sensitivity of 82.1%, and a specificity of 83.9%; this finding was true even in AFP-negative HCC subgroups, with an AUC of 0.831, a sensitivity of 81.2%, and a specificity of 83.2%. Additionally, serum levels of miR-21 were significantly associated with distant metastasis [97]. A meta-analysis study found serum miR-122 to be overexpressed in HCC patients with a pooled AUC of 0.82, a pooled sensitivity of 76%, and a pooled specificity of 75% [98,99]. The levels of serum exosomal miR-10b-5p and miR-215-5p were significantly higher in HCC patients, and serum exosomal miR-10b-5p could be a diagnostic biomarker for early-stage HCC with an AUC of 0.934, a sensitivity of 90.7%, and a specificity of 75.0% (cutoff value 1.8–fold) [102]. Serum exosomal miR-215-5p levels were significantly increased in patients with vascular invasion than in patients without vascular invasion and these higher levels were associated with poor prognosis in patients with HCC [102]. The expression of serum miR-424 was obviously decreased in HCC compared with healthy controls (AUC, 0.768), with a sensitivity and specificity of 75.0% and 72.4%, respectively [104]. Moreover, serum miR-424 levels were significantly correlated with metastasis and vein invasion. [104]. Additionally, circulating miRNA panels also showed good performance as early diagnostic biomarkers of HCC [22,105]. Thus, the combination of lncRNAs, circulating miRNAs, and AFP has great promise as a novel strategy with diagnostic and prognostic value for HCC.

### 3.4. Effect of lncRNA and miRNA Networks in Therapy Resistance

The aim of treatment is to prolong survival while maintaining the quality of life. The choice of treatment depends on the stage of diagnosis, commonly resection, ablation, and transplantation for early-stage HCC, transarterial chemoembolization for intermediate-stage HCC, and systemic treatments, such as tyrosine kinase inhibitors, chemotherapy agents, and immune checkpoint inhibitors, for advanced-stage HCC [83]. Only a small fraction of patients present with early-stage HCC, whose treatment is limited by the underlying liver disease. In recent years, systemic treatments have been available for advanced-stage HCC patients, but the resistance of agents remains a severe problem [106]. Many studies have indicated that lncRNA and miRNA networks are associated with drug resistance in HCC treatment (Table 4). 

The hepatitis B virus-encoded X (HBx) protein upregulated lncRNA TRERNA1 expression and enhanced sorafenib resistance in HBV-HCC. TRERNA1 acts as a ceRNA and sponges miR-22-3p; the potential target of miR-22-3p, NRAS, is then upregulated and activates the RAS/Raf/MEK/ERK signaling pathway [107]. HCC cells with high lncRNA HOTAIR expression enhanced sorafenib resistance by sponging miR-217 [112]. lncRNA MALAT1 is highly expressed in HCC sorafenib-resistant cells and upregulates the expression of Aurora-A by binding to miR-140-5p, thus enhancing sorafenib resistance [105]. Moreover, lncRNA TTN-AS1 [107], SNHG16 [113], and HANR [114] have also been shown to enhance sorafenib resistance. In contrast, the levels of lncRNA FOXD2-AS1 were decreased in sorafenib-resistant cells compared with those in the corresponding parental cells. Overexpression of FOXD2-AS1 upregulated transmembrane protein 9 (TMEM9) expression by binding FOXD2-AS1 to miR-150-5p, and increased sorafenib sensitivity [117]. Lenvatinib, as a multi-tyrosine kinase inhibitor, is also an option in first-line therapy for advanced HCC, but its resistance is still to be addressed. LncRNA MT1JP is overexpressed in lenvatinib-resistant cells; sponging miR-24-3p releases Bcl-2 like 2 (BCL2L2), which inhibits apoptosis and impairs the sensitivity of HCC cells to lenvatinib [118]. Olaparib is a poly ADP ribose polymerase (PARP) inhibitor. LncRNA DUXAP8 enhances resistance to olaparib by sponging miR-485-5p, upregulating forkhead box protein M1 (FOXM1) expression, and interacting with the RNA-binding protein fused in sarcoma (FUS) [119].

Resistance is a major challenge for chemotherapy agents. LncRNA UCA1 binds to miR-138-5p and activates the AKT/mTOR signaling pathway [120]. LncRNA NR2F1-AS1, which sponges miR-363, releases ATP binding cassette subfamily A member 1 (ABCA1) [121], which contributes to oxaliplatin resistance in HCC. Overexpression of lncRNA MALAT1 enhances doxorubicin resistance in HCC by mediating the miR-3129-5p/Nova1 axis [122]. lncRNA LEF1-AS1 combined with miR-10a-5p increases musashi1 (MSI1) expression and activates the AKT signaling pathway to stimulate cisplatin resistance in HCC [123]. Moreover, lncRNA LINC01234 enhances melanoma-associated antigen A3 (MAGEA3) expression by binding to miR-31-5p and increasing cisplatin resistance [125]. Nevertheless, upregulated lncRNA GAS5 could enhance cisplatin sensitivity by sponging miR-222 [124]. 

## 4. LncRNA and miRNA Networks in the Tumor Microenvironment of HCC

**Figure 2:**
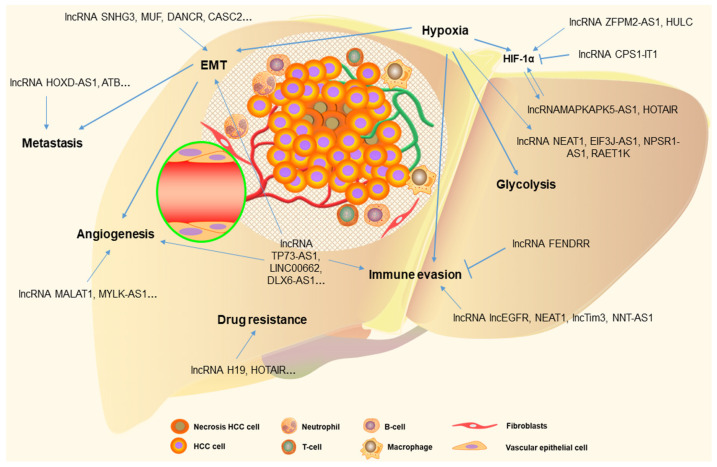
Long non-coding RNAs involved in the complex tumor microenvironment of HCC. LncRNA, long non-coding RNA; EMT, epithelial–mesenchymal transition; HIF-1α, hypoxia-inducible factor 1α.

The tumor microenvironment is complex, including that of HCC, involving crosstalk among tumor components such as HCC cells, the surrounding blood vessels, stromal cells, and immune cells, all of which are critical in tumorigenesis, angiogenesis, and metastasis. The immune checkpoint molecules, growth factors, cytokines, chemokines, noncoding-RNAs, and metabolites also participate in these interactions [126,127,128] (Figure 2).

Hypoxia is a crucial feature in solid tumors, including those in HCC, which induces a hypoxic response by upregulating hypoxia-inducible factors (HIFs), especially hypoxia-inducible factor 1α (HIF-1α). Furthermore, a cascade of metabolic changes in hypoxia facilitates cancer cell proliferation, glycolysis, EMT, drug resistance, and an immunosuppressive tumor microenvironment [129]. Recent studies have indicated that lncRNA and miRNA networks also play a key role in the metabolic changes associated with hypoxia. In HCC, highly expressed lncRNA zinc finger protein multitype 2 antisense RNA 1 (ZFPM2-AS1) [130] and HULC [131] bind to miR-576-3p and miR-377-5p, respectively, to upregulate the expression of HIF-1α. In addition, lncRNA MAPKAPK5-AS1 is significantly overexpressed in HCC and is directly activated by HIF-1α, which negatively regulates miR-154-5p to promote PLAG1 like zinc finger 2 (PLAGL2) expression. PLAGL2 activates the EGFR/AKT signaling pathway and increases the expression of HIF-1α; this positive signaling loop enhances hypoxia, HCC growth, and metastasis [132]. Hypoxia-induced lncRNAs also facilitate HCC proliferation and metastasis. Hypoxia-induced lncRNA NEAT1 [133] and EIF3J antisense RNA 1 (EIF3J-AS1) [134] promote HCC proliferation by regulating the miR-199a-3p/ uridine-cytidine kinase 2 (UCK2) and miR-122-5p/ catenin delta 2 (CTNND2) axis, respectively. HIF-1α activates the transcription of lncRNA retinoic acid early transcript 1 K (RAET1K), following the highly expressed lncRNA RAET1K sponging miR-100-5p to increase the expression of lactate dehydrogenase A (LDHA), which enhances glycolysis [135]. Overexpressed lncRNA HOTAIR targets miR-130a-3p to increase HIF-1α expression and glycolysis [136]. Moreover, hypoxia-inducible lncRNA neuropeptide S receptor 1 antisense RNA 1 (NPSR1-AS1) overexpression enhances the expression of p-ERK1/2 and pyruvate kinase M2 (PKM2) and activates the MAPK/ERK pathway to promote the growth and glycolysis of HCC cells [137]. In contrast to the abovementioned lncRNAs, lncRNA carbamoyl-phosphate synthetase 1 intronic transcript 1 (CPS1-IT1) levels were obviously decreased in HCC, while CPS1-IT1 overexpression reduced HIF-1α activation to inhibit EMT and metastasis [138]. 

Immune cells in the tumor microenvironment contain regulatory T cells (Treg), CD8+ T cells, CD4+ T cells, myeloid-derived suppressor cells (MDSCs), tumor-associated macrophages (TAMs), natural killer (NK) cells, and cancer-associated fibroblasts (CAFs), which contribute to inducing immune evasion, EMT, and drug resistance. Hypoxia induces an immunosuppressive tumor microenvironment, increasing the number of Treg and MDSC cells, promoting M2 macrophage polarization, and decreasing the number and activity of CD8+ cells and NK cells, which also aids in the immune evasion of tumor cells [129]. lncRNA fetal-lethal noncoding developmental regulatory RNA (FENDRR) inhibits the Treg-mediated immune escape of HCC cells by sponging miR-423-5p to upregulate the levels of growth arrest and the DNA-damage-inducible beta protein (GADD45B) [139]. In contrast, lncRNA lncEGFR stimulates Treg differentiation, inhibits cytotoxic T lymphocyte (CTL) activity in an EGFR-dependent manner, and promotes HCC immune evasion [140]. lncRNA NEAT1 is increased in the peripheral blood mononuclear cells (PBMCs) of patients with HCC, which decreases the antitumor activity of CD8 + T cells against HCC by regulating the miR-155/Tim-3 axis [141]. LncTim3 exacerbates CD8+ T cell exhaustion by specifically binding to Tim-3 [142]. In addition, overexpressed lncRNA TP73-AS1 sponges miR-539 to upregulate matrix metalloproteinase-8 (MMP-8) expression, which activates TGF-β1 signaling to induce M2 macrophage polarization in HCC [143]. LINC00662 competitively binds to miR-15a, miR-16, and miR-107 to promote Wnt-3a expression, following activation of the Wnt/β-catenin signaling pathway to promote M2 macrophage polarization [32]. HCC-derived exosomal lncRNA DLX6 antisense RNA 1 (DLX6-AS1) induces M2 macrophage polarization and promotes EMT by binding to miR-15a-5p to increase C-X-C motif chemokine ligand 17 (CXCL17) expression [144]. Exosomal lncRNA TUC339 is also involved in the regulation of macrophage polarization [145]. TAMs with M2 polarization contribute to HCC cell proliferation, angiogenesis, EMT, and interaction with other immune cells [146]. Additionally, TAMs are associated with CD8+ T cell conversion to an exhausted phenotype, which may be through the PD-1/PD-L1 and Tim3 signaling pathways [146]. 

In this complex tumor microenvironment, intercellular signaling communication plays an important role in the regulation of the biological behavior of HCC. Transforming growth factor-β (TGF-β) signaling is associated with inflammation, tissue fibrosis, cancer-related metastasis, angiogenesis, and immunosuppression, while activating other signaling pathways, such as extracellular signal-regulated kinase (ERK)/mitogen-activated protein kinase (MAPK) signaling and protein kinase B (Akt) signaling [147]. Overexpressed lncRNA NNT-AS1 decreases tumor CD4 lymphocyte infiltration by activating the TGF-β signaling pathway in HCC [148]. The lncRNA MEG8 promotes HCC progression via the miR-367-3p/14-3-3ζ/TGFβ-R1 axis [149]. LncRNA DLGAP1 antisense RNA 1 (D LGAP1-AS1) binds to miR-26a/b-5p to enhance the levels of interleukin-6 (IL-6), cyclin-dependent kinase 8 (CDK8), and low-density lipoprotein receptor-related protein 6 (LRP6), which activates the JAK2/STAT3 and Wnt/β-catenin signaling pathways, respectively, to facilitate tumorigenesis and EMT [150]. However, lncRNA TSLNC8 acts as a tumor inhibitor to suppress the IL-6/STAT3 signaling pathway [151]. Furthermore, lncRNA MALAT1 [152] targets miR-140 to upregulate VEGF-A expression and lncRNA MYLK Antisense RNA 1 (MYLK-AS1) [48] binds to miR-424-5p to increase the levels of E2F transcription factor 7 (E2F7) to activate the VEGFR-2 signaling pathway, which facilitates HCC progression and angiogenesis. 

## 5. Conclusions and Perspectives

A growing number of lncRNAs and miRNAs have been identified to play key roles in regulating HCC progression. The regulatory effect of miRNA occurs via binding to the 3′-UTR of target mRNAs, while lncRNAs are mainly sponges of miRNAs in the lncRNA and miRNA network, which is one of the critical lncRNA action modes, directly and indirectly, that modulates target gene expression, usually as the lncRNA–miRNA–mRNA axis. In the present review, we summarized the functional roles and related target genes of the lncRNA and miRNA networks. The dominant lncRNAs are oncogenic or suppressor lncRNAs in tumorigenesis and the progression of HCC. The dysregulated expression of lncRNAs contributes to clinical diagnosis and prognosis prediction as potential biomarkers. Additionally, lncRNA and miRNA networks are involved in the regulation of the tumor microenvironment, including EMT, hypoxia, glycolysis, and immune evasion. 

In the tumor microenvironment, hypoxia promotes glycolysis, EMT, and the formation of an immunosuppressive microenvironment. Simultaneously, the promotor lncRNAs could enhance hypoxia in a positive signaling loop, inducing M2 macrophage polarization to promote EMT and aggravating immune evasion. The interaction of tumor microenvironment components continues to deteriorate the microenvironment and facilitate HCC progression. However, there are limited studies on lncRNA inhibitors in the tumor microenvironment.

To date, most of the studies remain at the basic research stage, and little evidence regarding the clinical applications of lncRNAs or miRNAs has been presented (Table 5). As mentioned in the present review, lncRNA HOTAIR, MALAT1, and H19 play critical roles in HCC growth, angiogenesis, and resistance and may represent excellent therapeutic targets. However, as for miRNAs [153,154], a number of questions and challenges still exist for lncRNAs. First, in terms of on-target specificity, the efficacy of therapy may vary depending on the cell type. Second, efficient delivery, specific controlled delivery, and safety are the key points of lncRNA-based therapies. Lipid nanoparticles, polymers, RNA conjugations, and virus-based delivery have been used in clinically approved therapeutics or clinical testing. Additionally, exosome-mediated delivery, bacteriophages, and bacterial minicell delivery vehicles are still being developed. Third, efficacious doses and pharmacokinetics remain obstacles for development [155,156]. Further research and analyses of lncRNAs and miRNAs will provide more evidence and novel insights into the pathogenesis of HCC, which contributes to the diagnosis, therapy, and prognosis prediction of HCC.

## Figures and Tables

**Table 1:** Recent studies on the role of lncRNA and miRNA networks in hepatocellular carcinoma.

LncRNA	MiRNA	Target Gene of miRNA	Mechanism	References
**Promotor**				
MIAT	miR-214; miR-22-3p	EZH2, β-catenin; SIRT1	Proliferation, invasion	[23,24]
MCM3AP-AS1	miR-194-5p	FOXA1	Proliferation, cell cycle and apoptosis	[25]
HOXD-AS1	miR-130a-3p; miR19a	SOX4; ARHGAP11A	metastasis	[26,27]
HULC	miR-6825-5p, miR-6845-5p and miR-6886-3p	USP22	Autophagy	[28]
CDKN2BAS	miR-153-5p	ARHGAP18	Proliferation, metastasis	[29]
FAL1	miR-1236	AFP, ZEB1	Proliferation and migration	[30]
ATB	miR-200 family	ZEB1, ZEB2	Invasion, metastasis	[31]
LINC00662	miR-15a, miR-16, and miR-107	WNT3A	Proliferation, cell cycle, invasion	[32]
FLJ33360	miR-140	MMP9	Invasion, metastasis	[33]
LINC00958	miR-3619-5p	HDGF	Lipogenesis and progression	[34]
SNHG3	miR-128	CD151	Epithelial-mesenchymal transition (EMT)	[35]
H19	miR-193b	MAPK1	Invasion, migration	[36]
MUF	miR-34a	Snail1	EMT	[37]
NEAT1	miR-124-3p	ATGL	Proliferation	[38]
DANCR	miR-27a-3p; miR-214, miR-320a, and miR-199a	LIMK1; CTNNB1	Proliferation, EMT	[39,40]
CRNDE	miR-539-5p	POU2F1	Proliferation, migration, and invasion	[41]
AGAP2-AS1	miR-16-5p	ANXA11	Metastasis and EMT	[42]
CDKN2B-AS1	let-7c-5p	NAP1L1	Proliferation, metastasis	[43]
BACE1-AS	miR-377-3p	CELF1	Invasion and metastasis	[44]
SNHG11	miR-184	AGO2	Proliferation, migration, apoptosis, and autophagy	[45]
LINC01224	miR-330-5p	CHEK1	Proliferation and migration	[46]
PICSAR	miR-588	EIF6	Proliferation and apoptosis	[47]
MYLK-AS1	miR-424-5p	E2F7	Proliferation, invasion, metastasis, and angiogenesis	[48]
ST8SIA6-AS1	miR-129-5p	DCAF4L2	Proliferation, migration, and invasion	[49]
SNHG16	miR-605-3p	TRAF6	Metastasis and EMT	[50]
OSER1-AS1	miR-372-3p	Rab23	Proliferation, migration, and invasion	[51]
LINC00460	miR-342-3p	AGR2	Proliferation, metastasis	[52]
LINC00668	miR-532-5p	YY1	Proliferation, migration, invasion and EMT	[53]
MALAT1	miR-125a-3p	FOXM1	Proliferation and metastasis	[54]
Linc00152	miR-193a/b-3p	CCND1	Proliferation	[55]
**Suppressor**				
CASC2	miR-367	FBXW7	EMT	[56]
EPB41L4A-AS2	miR-301a-5p	FOXL1	Proliferation, migration, and invasion	[57]
TCL6	miR-106a-5p	PTEN	Proliferation	[58]
MEG3	miR-544b	BTG2	Proliferation, migration, and invasion	[59]
MAGI2-AS3	miR-374b-5p	SMG1	Proliferation and migration	[60]
LINC01018	miR-182-5p	FOXO1	Proliferation and apoptosis	[61]
DGCR5	miR-346	KLF14	Proliferation, migration, and invasion	[62]
MIR31HG	miR-575	ST7L	Proliferation and metastasis	[63]
LINC01488	miR-124-3p/miR-138-5p	vimentin	Metastasis and tumorigenesis	[64]
LINC00657	miR-106a-5p	PTEN	Proliferation, migration, and invasion	[65]
TUSC7	miR-10a	EphA4	EMT	[66]
MIR22HG	miR-10a-5p	NCOR2	Proliferation, migration, and invasion	[67]

Abbreviations: LncRNA, long non-coding RNA; miR, MicroRNA.

**Table 2:** Recent studies on the expression levels of potential lncRNA-based biomarkers in hepatocellular carcinoma.

LncRNA	Expression	Detectable Location	Background	Biomarker Category	References
ZFAS1	upregulated	plasma	-	diagnosis	[86]
LINC00853	upregulated	serum extracellular vesicle	-	diagnosis	[87]
TSPAN12	upregulated	tissues	HCC with MVI	diagnosis/prognosis	[88]
SEMA6A-AS1	downregulated	tissues	HBV-HCC	prognosis	[89]
SOX2OT	upregulated	tissues	-	prognosis	[90]
UCA1	upregulated	serum, tissues	-	diagnosis/prognosis	[91]
WRAP53	upregulated	serum, tissues	-	diagnosis/prognosis	[91]
DGCR5	downregulated	serum, tissues	-	diagnosis/prognosis	[78]
PANDAR	upregulated	tissues	-	prognosis	[92]
HOTTIP	upregulated	tissues	-	prognosis	[93]
LINC00161	upregulated	serum, exosome	-	diagnosis	[94]
LOC101926913	upregulated	tissues	-	prognosis	[95]
LINC00152	upregulated	serum	-	diagnosis	[72]
NONHSAT053785	upregulated	serum	-	diagnosis	[96]

Abbreviations: LncRNA, long non-coding RNA; MVI, microvascular invasion.

**Table 3:** Recent studies on the expression levels of potential circulating miRNA biomarkers in hepatocellular carcinoma.

MiRNA	Expression	Detectable Location	Background	Biomarker Category	References
miR-21	upregulated	serum	-	diagnosis	[97]
miR-122	upregulated	serum	-	diagnosis/prognosis	[98,99]
miR-665	upregulated	serum	-	prognosis	[100]
miR-148a	downregulated	plasma	-	diagnosis	[101]
miR-10b-5p	upregulated	serum exosome	-	diagnosis	[102]
miR-215-5p	upregulated	serum exosome	-	prognosis	[102]
miR-126	upregulated	plasma	HBV-HCC	diagnosis	[103]
miR-424	downregulated	serum	-	diagnosis/prognosis	[104]

Abbreviations: miR, MicroRNA.

**Table 4:** LncRNA and miRNA networks in hepatocellular carcinoma therapy resistance.

Drug	LncRNA	Resistance	Mechanism	References
Sorafenib	Translation Regulatory Long Non-Coding RNA 1 (TRERNA1)	enhance	miR-22-3p/NRAS axis	[107]
	Nicotinamide nucleotide transhydrogenase antisense RNA 1 (NNT-AS1)	enhance	miR-16-5p/cyclin E1	[108]
	Kcnq1 overlapping transcript 1 (KCNQ1OT1)	enhance	miR-506/ PD-L1	[109]
	POIR	enhance	sponging miR-182-5p	[110]
	H19	enhance	upregulating miR-675	[111]
	HOX transcript antisense intergenic RNA (HOTAIR)	enhance	sponging miR-217	[112]
	Small nuclear RNA host gene 16 (SNHG16)	enhance	miR-23b-3p/ EGR1	[113]
	MALAT1	enhance	miR-140-5p/Aurora-A	[114]
	HCC associated long non-coding RNA (HANR)	enhance	miR-29b/ATG9A axis	[115]
	Nuclear paraspeckle assembly transcript 1 (NEAT1)	enhance	miR-204/ATG3	[116]
	FOXD2 Adjacent Opposite Strand RNA 1 (FOXD2-AS1)	weaken	miR-150-5p/ TMEM9	[117]
	Small nucleolar RNA host gene 3 (SNHG3)	enhance	miR-128/CD151	[35]
Lenvatinib	Metallothionein 1 J, pseudogene (MT1JP)	enhance	miR-24-3p/BCL2L2 axis	[118]
Olaparib	Double homeobox A pseudogene 8 (DUXAP8)	enhance	miR-485-5p/ FOXM1	[119]
Oxaliplatin	UCA1	enhance	sponging miR-138-5p	[120]
	Nuclear receptor subfamily 2 group F member 1-antisense RNA 1 (NR2F1-AS1)	enhance	miR-363/ABCC1	[121]
Doxorubicin	MALAT1	enhance	miR-3129-5p/Nova1 axis	[122]
Cisplatin	Lymphoid enhancer-binding factor 1 antisense RNA 1 (LEF1-AS1)	enhance	miR-10a-5p/MSI1	[123]
	Growth-arrest-specific transcript 5 (GAS5)	weaken	sponging miR-222	[124]
	LINC01234	enhance	miR-31-5p/MAGEA3	[125]

Abbreviations: LncRNA, long non-coding RNA; miR, MicroRNA.

**Table 5:** LncRNA-based or miRNA-based therapy clinical trials in cancer.

Registration Number	Phase	Regimen	Target	Target Population	Result	Reference
NCT03719300	Phase 2	BC-819	lncRNA H19	Non-muscle Invasive Bladder Cancer	Lack of efficacy	
NCT01829971	Phase 1	MRX34	miR-34	Primary Liver Cancer, SCLC, Lymphoma, Melanoma, Multiple Myeloma, Renal Cell Carcinoma, NSCLC	Early terminated (Five immune related serious adverse events)	[157]
NCT02369198	Phase 1	TargomiRs	miR-16	Malignant Pleural Mesothelioma, NSCLC	ORR: 5%, SD: 68%	[158]
NCT02580552	Phase 1	Cobomarsen	miR-155	Cutaneous T-cell Lymphoma, Mycosis Fungoides, Chronic Lymphocytic Leukemia, Diffuse Large B-Cell Lymphoma (ABC Subtype), Adult T-Cell Leukemia/Lymphoma	Completed	
NCT03713320	Phase 2	Cobomarsen	miR-155	Cutaneous T-Cell Lymphoma/Mycosis Fungoides	Early terminated for business reasons, and not due to concerns regarding safety or lack of efficacy	

Abbreviations: LncRNA, long non-coding RNA; miR, MicroRNA; NSCLC, non-small cell lung cancer; ORR, objective response rate; SCLC, small-cell lung cancer; SD, stable disease.

## Data Availability

Not applicable.
